# A visible, targeted high-efficiency gene delivery and transfection strategy

**DOI:** 10.1186/1472-6750-11-56

**Published:** 2011-05-21

**Authors:** Qiao-Ying Yuan, Jing Huang, Bao-Cheng Chu, Xing-Sheng Li, Liang-Yi Si

**Affiliations:** 1 Department of Geriatrics, Southwest Hospital, the Third Military Medical University, Chongqing 400038, China; 2 Institute of Ultrasound Imaging, The Chongqing University of Medical Sciences, Chongqing 400010, China; 3 BioMolecular Imaging Center, Department of Radiology, University of Washington, Seattle, WA 98109, USA

**Keywords:** Intramyocardial delivery, angiogenic gene, microbubbles, gene expression, angiogenesis

## Abstract

**Background:**

To enhance myocardial angiogenic gene expression, a novel gene delivery strategy was tested. Direct intramyocardial injection of an angiogenic gene with microbubbles and insonation were applied in a dog animal model. Dogs received one of the four different treatments in conjunction with either the enhanced green fluorescence protein (EGFP) gene or the hepatocyte growth factor (HGF) gene: gene with microbubbles (MB) and ultrasound (US); gene with US; gene with MB; or the gene alone.

**Results:**

Distribution of MB and the gene in the myocardium was visualized during the experiment. Compared with the EGFP gene group, an average 14.7-fold enhancement in gene expression was achieved in the EGFP+MB/US group (P < 0.01). Compared with the HGF gene group, an average 10.7-fold enhancement in gene expression was achieved in the HGF+MB/US group (P < 0.01). In addition, capillary density increased from 20.8 ± 3.4/mm2 in the HGF gene group to 146.7 ± 31.4/mm2 in HGF+MB/US group (P < 0.01).

**Conclusions:**

Thus, direct intramyocardial injection of an angiogenic gene in conjunction with microbubbles plus insonation synergistically enhances angiogenesis. This method offers an observable gene delivery procedure with enhanced expression efficiency of the delivered gene.

## Background

Recently, delivering biological agents to areas of the myocardium to treat cardiovascular disease has attracted much attention [1]. The delivery of angiogenic genes to the myocardium is one such approach. For example, angiogenesis was reported to occur with direct intramyocardial injection of a plasmid vector during open-heart surgery in patients [2]. However, compared with viral vectors, the gene expression for intramyocardial injection of nonviral vectors is less efficient. Hepatocyte growth factor (HGF) acts as a potent cell growth factor and promotes angiogenesis of the infracted myocardium [3,4]. Thus, HGF could improve angiogenesis in the chronically ischemic myocardium, which indicates a potent therapeutic value of HGF gene transfection for treating chronic ischemic heart diseases [5,6]. Real-time magnetic resonance imaging has been used to monitor the delivery of therapeutic reagents to the myocardium. However, its clinical application is limited by its potential side effects and complications [7]. Therefore, an effective and safe delivery strategy is needed to enhance therapeutic angiogenic responses in ischemic myocardium.

Recently, some studies have showed that microbubbles can be mixed with DNA and then be delivered to targeted areas [8-10]. Microbubbles (MBs) can lower the energy threshold of US for cavitation, which transiently perforates the cell membrane or disrupts the capillary wall to allow delivery of bioactive agents into cells or the interstitial space; many studies of microbubble applications combined with insonation in rodents are emerging [11-17]. Whether regional application of an angiogenic gene and microbubbles combined with ultrasound in bigger animals would enhance gene expression and angiogenesis is not known. Moreover, microbubbles may be also used as an imaging marker to show the distribution of the therapeutic agents mixed with microbubbles in the myocardium. Therefore, it is of interest to further investigate the regional application of microbubbles in larger animals.

EGFP has been widely used as a reporter gene [18]. In the first part of this study, we tested the feasibility of direct intramyocardial delivery of the EGFP gene and microbubbles to enhance gene expression by measuring the EGFP expression in the normal myocardium of dogs. We next evaluated the gene expression and the angiogenic effects of direct intramyocardial delivery of a mixture of a low-dose of the HGF gene and microbubbles combined with insonation in dogs with induced myocardial infarction. We found this technique to be a promising, visible, and effective intramyocardial gene delivery system.

## Materials and methods

### Animal procedures

Male adult hybrid dogs (12-15 kg) from the Laboratory Animal Center of Chongqing University of Medical Science were used in this study. All experimental procedures conformed to the Guide for the Care and Use of Laboratory Animals published by the US National Institutes of Health (NIH publication 85-23, revised 1996). Sixteen dogs were randomly divided into four groups (n = 4) to receive the following treatments: (1) EGFP, microbubbles, and ultrasound (EGFP+MB/US group); (2) EGFP and ultrasound (EGFP+US group); (3) EGFP and microbubbles (EGFP+MB group), and (4) EGFP alone. Acute myocardial infarction was induced in another 36 dogs, which then were divided into six groups of six animals and treated with HGF+MB/US, HGF+US, HGF+MB, HGF alone, MB/US, or surgery alone (control). All procedures on animals were approved by the Institutional Animal Care and Use Committee at Chongqing University of Medical Science.

### Preparation of microbubbles

Microbubbles were prepared at the Institute of Ultrasound Imaging at Chongqing Medical University as described by Li et al. (2008) [12]. The mean diameter and concentration of the microbubbles were measured using a Coulter counter (Coulter Electronics, Hialeah, FL, USA). The microbubble solution was sterilized by 60Co irradiation. The quantified plasmids (pEGFP) then were added and mixed gently with the microbubbles. The microbubble solution was then diluted with saline to a final concentration of 1.42 ± 0.26 ×109/ml before intramyocardial injection. The diameter range of the self-made lipid microbubble was 2.11 to 6.43 μm; the mean diameter was 2.79 μm.

### Plasmid purification

Plasmid DNA was produced according to a standard method (TaKaRa Biotechnology, Tokyo, Japan) and purified to remove endotoxin contamination. The plasmids pEGFP-N2 and pcDNA3.0-HGF were kindly provided by Dr. Zuo YL and Li XS of Chongqing University of Medical Science, China. The plasmid contained the EGFP -N2 cDNA that uses a CMV promoter to drive EGFP expression, we used a naked HGF plasmid (AnGes MG Inc., Tokyo, Japan), which contained the human HGF complementary DNA (2.2kb) that uses the cytomegalovirus promoter/enhancer to drive HGF expression. Purified plasmid DNA was dissolved in sterile water and stored at -20°C.

### Plasmid pEGFP transfer

After an abdominal cavity injection of sodium pentobarbital (15 mg/kg), the heart of each of the 16 dogs in the EGFP experiment was exposed by median sternotomy. For the dogs in the EGFP+US/MS group and the EGFP+MB group, 500 μg of total plasmid DNA in 0.5 ml of microbubble solution were injected intramuscularly at the left anterior free wall at five separate sites. For the ultrasound treatment, an ultrasound transducer then was placed on the heart separated by a 2 mm ultrasonic coupling agent. In the EGFP+MB/US group, continuous insonation was provided using an ultrasound gene transfection treatment meter [UGT]-1025 (Institute of Ultrasound Imaging of Chongqing Medical University, Chongqing, PR China) under the following conditions: 30 s with a 10 s pause, 1 MHz, 1 W/cm2, 60 s in total at each site [19]. In the EGFP+US group, the same amount of the EGFP gene without MB was injected into the myocardium, followed by the same insonation procedure. Only the gene was injected into the myocardium in the EGFP group without MB and US. An electrocardiogram was continuously monitored during the process. After these procedures, the animals were allowed to recover.

### Production of acute myocardial infarction

The remaining 36 dogs were used to generate the myocardial infarction model. They were intraperitoneally anesthetized with sodium pentobarbital (15 mg/kg), then intubated and ventilated with a volume-cycled small-animal ventilator. An anterior thoracotomy was performed to open the pericardium. The heart was then rapidly exteriorized, and a 4-0 silk suture was tightened around the proximal left anterior descending coronary artery (before the second branch of the diagonal artery). Visible epicardial feeders at the margins of the occluded bed were ligated to decrease the collateral and produce transmural injury [20].

Aqueous penicillin G (200 000U/Kg) was given the day of, and the day after surgery as prophylaxis against wound infection. The incision remained dry for all dogs. The dogs were provided an appropriate environment including a clean and warm shelter and a comfortable resting area, ready access to fresh water and a diet to maintain full health and vigour. Zubrin tablets (Tepoxalin, 100 mg tablets, Schering-Plough Animal Health Corp) were administered in moist food for five days. Dogs received 20 mg/kg once a day on the initial day of treatment followed by a daily maintenance dose of 10 mg/kg once a day. We found that the dogs' average recovery to normal activity was five days after surgery. The dogs were divided into the following six treatment groups after the models of myocardial infarction were prepared (n = 6): (1) HGF+MB/US; (2) HGF+US; (3) HGF+MB; (4) HGF alone; (5) MB/US; and (6) surgery alone (control).

### Plasmid pcDNA3.0-HGF transfer

Five minutes after the coronary artery was tightened, 500 μg of total plasmid DNA in 0.5 ml of lipid microbubble solution were injected along the left ventricular free wall at five separate sites in the dogs in the HGF+MB/US group and the HGF+MB group. Then, continuous insonation was administered via an ultrasound gene transfection treatment meter [UGT]-1025 for 30 s with a 10s pause at 1-MHz and 1-W/cm2 for 60s in total at each site in the HGF+MB/US group [19]. In the HGF+US group, the HGF gene was injected into the myocardium, followed by the same insonation procedure. In the MB/US group, the MB was injected into the myocardium, followed by the same insonation procedure. In the HGF alone group, only the gene was injected. In the control group, 0.5 ml of saline was injected into the myocardium. An electrocardiogram was continuously monitored during the process. After these procedures, the animals were allowed to recover.

### Analysis of regional microbubble imaging

After intramyocardial injection of a mixture of 100 μg of pEGFP and 0.1 ml of microbubble solution, ultrasonic gel (Aquasonics 100; Parker Labs, USA) was administered around the injection site of three new dogs; transthoracic diagnostic echocardiography (a Sonos 4500 ultrasound system and a 3 MHz linear array transducer, Philips Inc., USA) was used to measure the area of microbubbles existence in the regional myocardium at the time points 0, 5, 10, 20, 30, 40, 50, and 60 min.

### Histopathological examination and analysis of creatine kinase (CK)

The 16 dogs in the EGFP experiment were deeply anesthetized with pentobarbital at 48 h, and the heart was rapidly excised. After removal of the atrium and the right ventricle, the left ventricle was opened and examined to determine the gross appearance of the specimen. Cross-sectional surfaces at the injection sites were also inspected for intramural hematomas. Following macroscopic examination, the hearts were transversally sliced. Part of the myocardium was fixed in formalin. Paraffin-embedded myocardium was cut into 5-7 μm thick slices. CK secretion was measured to test for myocardial damage using a Hitachi 7170A automatic biochemistry analyzer (Japan). CK secretion was measured before the experiment, during the operation, at the end of the experiment, and at 24h and 48 h after transfection in the four EGFP groups.

### Evaluation of EGFP gene expression

EGFP gene expression was assessed at 48 h after transfection. EGFP mRNA levels were monitored by RT-PCR using a two-step RT-PCR kit (AMV, TaKaRa, Japan) on a PCR system (MyCycler, BioRad). Sequences of primers for EGFP were: 5'- GCC ACA AGT TCA GCG TGT C -3' (sense), 5'- TCA CCT TGA TGC CGT TCT T -3' (antisense), and RT-PCR was performed under the following conditions: an initial heating to 94°C for 2 min; then 94°C for 30 s, 55°C for 40 s, 72°C for 40 s for 38 cycles; and then 72°C for 5 min. The products were separated by gel electrophoresis. All electrophoresis blots were quantified using Quantity One software (BioRad).

### Evaluation of EGFP protein expression

EGFP protein expression was assessed using a laser scanning confocal fluorescence microscope (Carl Zeiss LSM510, Axiovert 100 M, Jena, Germany). After the dogs were sacrificed, the heart was excised and placed in optimum cutting temperature compound and shock frozen prior to storage at -20°C. The fluorescence was visualized at 488nm excitation wavelengths using a confocal laser scanning fluorescence microscope (Carl Zeiss LSM510, Axiovert 100 M, Jena, Germany). Each sample measurement was repeated eight to ten times.

### Evaluation of HGF gene expression

HGF gene expression was assessed at day 28 after transfection. The expression of HGF was assessed using commercially available ELISA kits (Duoset HGF ELISA DY-294; R&D Systems Inc., USA) following the protocols recommended by the manufacturer. Briefly, enzyme-linked polyclonal antibodies specific for HGF protein were added to the wells, and the intensity of the color was measured and compared with a standard curve.

### Evaluation of capillary density

Paraffin-embedded myocardium was cut into 3-4 μm thick slices. Specimens were incubated with polyclonal rabbit anti-dog CD31 antibody (platelet endothelial cell adhesion molecule 1; bs-0468R, Beijing Biosynthesis Biotechnology Co. Ltd., Beijing, PR China) at 4°C overnight. The number of capillaries was counted in regions with transversely sectioned myocytes in the border zone. Five fields per section were randomly selected and analyzed. Twenty sections per heart were blindly evaluated to estimate capillarity. The number of capillaries was assessed from photomicrographs using a computerized image analysis system (Image Pro Plus 4.0, Media Cybernetics. L. P., USA).

### Detection of myocardial perfusion

At day 28 after transfection, the myocardial infarction-induced dogs treated with HGF were reanesthetized and their chests were opened. Approximately 700,000 radioactive microspheres (NEN Life Science Product, Inc., Boston, MA, USA) labeled with 51Cr were injected into the left ventricular cavity [21]. The microspheres were allowed to circulate for 1 min. The animals were then euthanized and their hearts were excised. Tissue specimens from the left ventricle, including the septum and right ventricular free wall, were excised for measurement of radioactivity. The activities in the left ventricle were expressed per weight of heart tissue as a ratio relative to the activity in the right ventricular free wall [21].

### Statistical analysis

Data are expressed as mean ± standard deviation (sd). Analysis of variance (SPSS 14.0 Statistical Software, SPSS, Inc., Chicago, IL, USA) was used to assess the capillary density, protein expression, and EGFP and HGF mRNA levels. Viability comparisons across multiple experimental groups were conducted using a one-way analysis of variance (ANOVA) followed by post-hoc Tukey analysis to determine significant differences. P < 0.05 was considered to be statistically significant.

## Results

A total of 40 animals were subjected to coronary artery ligation; none of the animals died during the operation. 38 (95%) survived and were included in the experiments. Although two dogs died at during 24 hour after the treatments, no dogs died subsequently until the final evaluation at four weeks. The dogs with measured infarct sizes less than 20% of LV circumference were excluded from the study (one dog in MI group, one dog in HGF-US/MB group).

### Gross pathology

No acute pericardial tamponade or episodes of sustained ventricular arrhythmia were noticed after intramyocardial injection of microbubbles and a gene in the animals. The pericardial sac was examined, and pericardial effusion and hematoma were not observed. Harvested hearts had no intramyocardial hematoma, splits, or perforations.

### CK secretion

As shown in Table 1, there was no significant difference in CK secretion among the four EGFP groups (i.e., among measurements taken before the experiment, during the operation, at the end of the experiment, and at 24 and 48 h after transfection).

**Table 1:** Analysis of the serum CK activity in the four groups ( ± sd, n = 4)

CK(U/L)	EGFP+MB/US	EGFP+US	EGFP+MB	EGFP alone
Experiment before	269 ± 59	284 ± 62	279 ± 64	258 ± 56
During the operation	271 ± 61	291 ± 66	281 ± 70	261 ± 55
End of the experiment	278 ± 60	288 ± 60	284 ± 63	268 ± 61
24 h	264 ± 58	282 ± 62	277 ± 61	267 ± 59
48 h	268 ± 54	279 ± 59	275 ± 60	253 ± 57

### Microbubble imaging characterization

**Figure 1:**
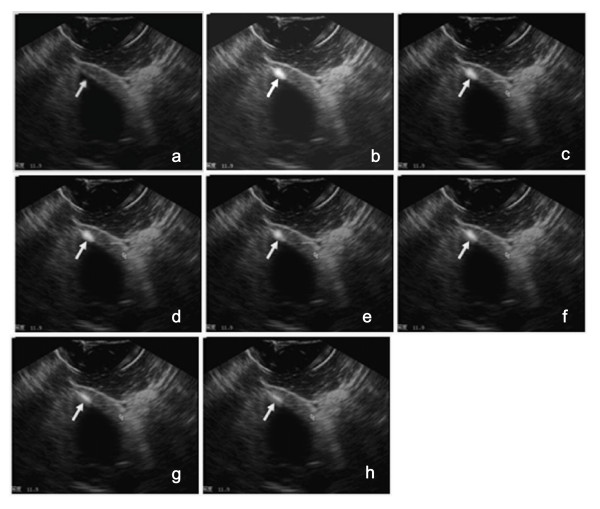
**Imaging of the microbubbles at the intramyocardial injection site**. EGFP plasmid DNA (100 μg) was mixed with 0.1 ml of microbubble solution and injected into the myocardium of the left ventricle anterior wall. (a) Long-axis view before contrast injections. (b) The same long-axis view showing contrast injection at 5 min (indicated by the white arrow); the contrast is visible as the hyperintense signal indicated by the white arrow. (c) Long-axis view showing contrast injection at 10 min. (d) Long-axis view showing contrast injection at 20 min. (e) Long-axis view showing contrast injection at 30 min. (f) Long-axis view showing contrast injection at 40 min. (g) Long-axis view showing contrast injection at 50 min.(h) Long-axis view showing contrast injection at 60 min.

Long-axis images confirmed the presence of microbubbles and the gene at the injection sites, as seen in ultrasound images acquired in real time (Figures 1a, 1b, 1c, 1d, 1e, 1f, 1g, 1h). The distribution and the sizes of the microbubble mixture in the myocardium were clearly observed once injection was completed. The microbubble-enhanced imaging regions were mainly confined to approximately one half of the myocardial thickness (Figure 1b). Clear imaging of intramyocardial injection of 0.1 ml diluted microbubbles at a concentration of 1.42 ± 0.26 ×109/ml was possible.

### Area of the microbubble-enhanced imaging regions

The extent of intramyocardial microbubble-enhanced imaging (maximal depth and width) was measured to identify local diffusion of the microbubble tracer at the injection site. The imaging sizes of the mixture of microbubbles and the EGFP gene in regional myocardium was measured in three dogs using a Sonos 4500 ultrasound system. When the needle was extended 6 mm into the myocardium and the injection volume of microbubbles and the gene was 0.1 ml, the imaging size was 3.2 ± 1.0 mm in depth and 7.6 ± 1.9 mm in maximal width at the moment of injection. The imaging realm was 1.8 ± 0.6 mm in depth and 4.3 ± 1.1 mm in maximal width after 60 min. The area of the contrast-enhanced region of 0.1 ml microbubbles and the gene decreased from 24 ± 2 mm2 initially to 10 ± 1 mm2 after 60 min (P < 0.05)

### Imaging persistence of microbubbles

Visualization of microbubbles and the gene at the injection site was distinct. The image intensity gradually decreased in the myocardium. The gray-scale intensity of the microbubble-enhanced regions changed slowly and slightly within 30 min, and then the intensity gradually decreased. The image intensity and extent faded by about 60-70% after 60 min (Figure 1h), but the microbubble-enhanced imaging regions in the myocardium of the left ventricle free wall were still visible at this time (Figure 1h).

### Detection of EGFP mRNA expression

**Figure 2:**
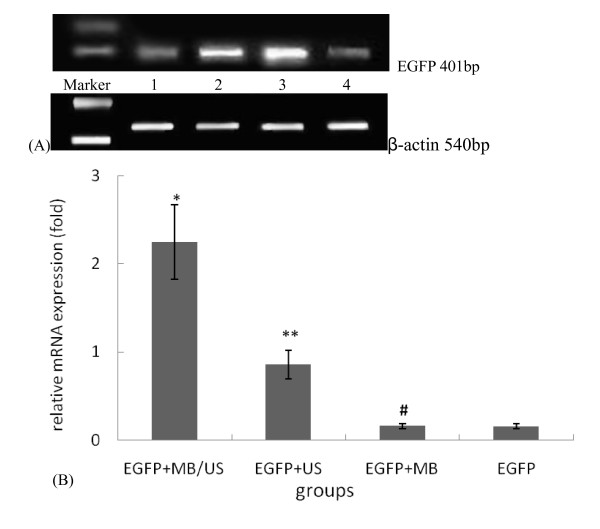
**EGFP mRNA expression in injection regions of the myocardium for four different treatment groups, as evaluated using RT-PCR technology**. ( ± sd, n = 4). (a)M: marker; 1 lane: EGFP group; 2 lane: EGFP + US group; 3 lane: EGFP +MB/US group; 4 lane: EGFP +MB group. (b) *P < 0.01 vs. EGFP group and EGFP+ MB group; **P < 0.01 vs. other three groups. # P > 0.05 vs. the EGFP group. β-actin was used as the internal control.

RT-PCR data revealed expression of EGFP mRNA in all groups (Figure 2a). EGFP was expressed at low levels in the EGFP group, and no statistical differences in EGFP expression were detected between the EGFP+MB group and the EGFP group. The EGFP mRNA expression of the EGFP+MB/US group was about 14.7 times higher (P < 0.01) and the EGFP+US group was about 5.6 times higher (P < 0.01) than that of the EGFP alone group (Figure 2b).

### Detection of EGFP protein expression

**Figure 3:**
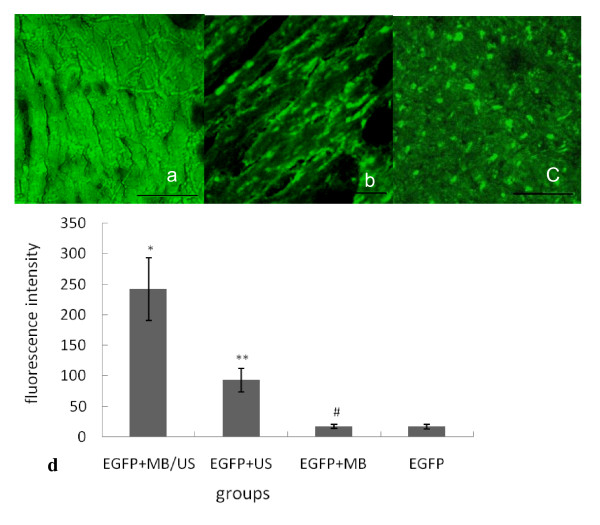
**EGFP protein expression in injection regions of the myocardium for four different treatment groups, as evaluated using confocal laser scanning microscopy**. (Scale bar = 50 μm). (a) Expression of EGFP in the myocardium of the EGFP+MB/US group (original magnification ×400). (b) Expression of EGFP in the myocardium of the EGFP+US group (original magnification ×400). (c) Expression of EGFP in the myocardium of the EGFP group (original magnification ×400). (d) ( ± sd, n = 4). *P < 0.01 vs. the other three groups; **P < 0.01 vs. the EGFP group and the EGFP+MB group; # P > 0.05 vs. the EGFP group.

Confocal laser scanning fluorescence microscopy revealed that green fluorescence was observed throughout the myocardial cells around the injection region in the EGFP+MB/US group after 48 h (Figures 3a, 3b, 3c). As shown in Figure 3d, the EGFP fluorescence intensity of the EGFP+MB/US group was about 11.6 times higher (P < 0.01) and that of the EGFP +US group was about 3.9 times higher (P < 0.01) than that of the EGFP alone group. In the EGFP alone group, weak green fluorescence could be seen in scattered cells within the myocardium, and its intensity was obviously lower than that in the EGFP+MB/US group and the EGFP+US group. There was no significant difference in green fluorescence between the EGFP+MB and the EGFP groups (P > 0.05).

### Human HGF gene expression in ischemic myocardial muscles

**Figure 4:**
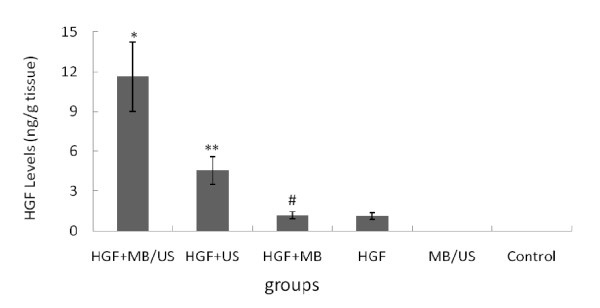
**Analysis of HGF content in the myocardium as measured by ELISA for four different treatment groups**. HGF protein expression was assessed at day 28 after transfection ( ± sd, n = 6). *P < 0.01 vs. other five groups; **P < 0.01 vs. HGF +MB group and HGF alone group; # P > 0.05 vs. HGF alone group.

ELISA data showed that the HGF content in the myocardium was the highest in the HGF+MB/US group (Figure 4) (P < 0.01). There was no significant difference between the HGF+MB and the HGF groups (P > 0.05).

### Capillary density

**Figure 5:**
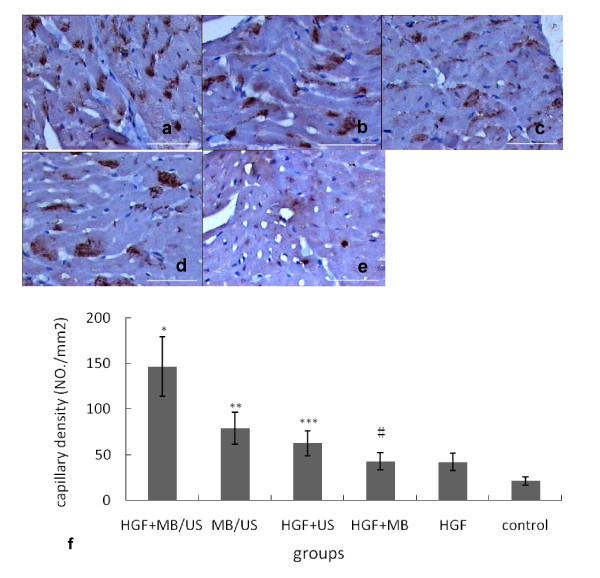
**Immunohistochemistry results of CD31 expression in five different treatment groups**. The number of blood vessels in transverse sections of the dog ischemic myocardium harvested at day 28.(Scale bar = 20 μm). (a) Expression of CD31 in the HGF+MB/US group (original magnification ×400). (b) Expression of CD31 in the MB/US group (original magnification ×400). (c) Expression of CD31 in the HGF+US group (original magnification ×400). (d) Expression of CD31 in the HGF group (original magnification ×400). (e) Capillaries in the control group. (f) The capillary density of HGF+MB/US group was the highest among all groups ( ± sd, n = 6). *P < 0.01 vs. other five groups; **P < 0.01 vs. HGF +US group, HGF alone and HGF +MB group; *** P < 0.01 vs. HGF alone and HGF +MB group; # P > 0.05 vs. HGF alone group.

CD31 antibody was used to detect capillaries at day 28 post transfection in the HGF-treated dogs. Immunohistochemistry results showed that CD31 expression appeared as brown granules, which were located on the membrane and endochylema of vascular endothelial cells. The capillary density in the HGF+MB/US group was the greatest (Figure 5). The increased capillary density was mainly limited to the area around the infarct area (border zone). Capillary densities of the HGF+MB/US group (146.7 ± 31.4/mm2) and the HGF+US group (62.5 ± 16.4/mm2) were significantly higher (P < 0.01) than that of the HGF+MB group (42.6 ± 11.9/mm2), the HGF group (41.9 ± 10.5/mm2), and the control group (20.8 ± 3.4/mm2). The capillary density was (78.7 ± 23.4/mm2) in the MB/US group. There was no significant difference in the capillary density between the HGF+MB and the HGF groups (P > 0.05).

### Measurement of regional blood flow

**Figure 6:**
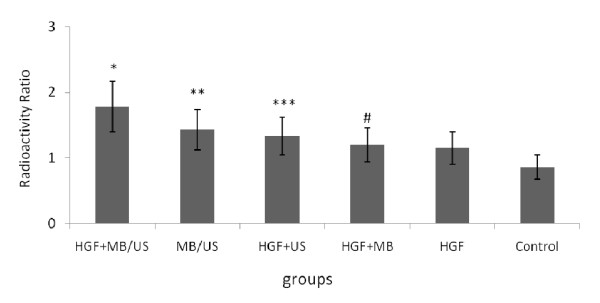
**Effect of intramyocardial injection of HGF/microbubbles combined with ultrasound on regional myocardial blood flow**. ( ± sd, n = 6). The radioactivities in the left ventricle were expressed per weight of heart tissue as a ratio relative to the activity in the right ventricular free wall. *P < 0.01 vs. other five groups; **P < 0.01 vs. HGF +US group, HGF alone and HGF +MB group; ***P < 0.01 vs. HGF alone and HGF +MB group; # P > 0.05 vs. HGF alone group.

Radioactive microspheres were used to measure relative blood flow. The right ventricular free wall served as the reference region. The average weights of the left ventricle and the right ventricular free wall in each heart were 77.6 g and 12.9 g, respectively. The regional blood flow ratio was significantly higher in the HGF-treated dogs than in the control group (Figure 6). The regional blood flow ratio was highest in the HGF+MB/US group.

## Discussion

Previous animal studies have proven the feasibility of enhancing collateral function by delivery of angiogenic factors to the myocardium [1,2,22-24]. Intramyocardial gene transfer allows nonviral vector delivery directly to the target area. Plasmid delivery methods are relatively safer, but their transfection efficiencies are low even after direct injection. Recently, researchers have begun to use microbubble contrast agents as a diagnostic imaging tool and as a gene carrier [11-14]. Some studies have shown that ultrasound, used either alone or in combination with microbubbles, can delivery of genes to specific tissues, including the myocardium and increase gene expression [25-28].

Systemic administration of HGF by ultrasound-targeted microbubble destruction for therapeutic angiogenesis has been demonstrated to be effective [3-6]. More, US/MB has been used to enhance the delivery of HGF into the skeletal muscle by direct intramuscular injection of a naked plasmid into the rat hind limbs [27]. This technique was applied to the heart, and the successful cardiac delivery of a reporter gene was achieved by using an adenovirus vector loaded on MBs [25]. However, for the therapeutic use in patients, innovations in non-viral plasmid DNA-based gene transfer with high transfection efficiency would be desirable [11]. In this study, when US/MB was combined, naked HGF plasmid injected into the blood circulation, not the direct injection or intracoronary injection, was successfully transferred into the myocardium. These results provide the basis for the application of US/MB for non-viral transfection of therapeutic genes into the myocardium by systemic administration of the naked plasmid [13-15]. However, the naked plasmid injected into the blood is easily degraded by DNase or captured by non-specific scavenger receptors [11,29], which leads to rapid decrease in concentration of the plasmid reaching the target regions. Thus a high dose of the plasmid could be needed for intravenous injection to achieve the similar plasmid concentration obtainable with intramyocardial injection [11]. Therefore, further studies are necessary to optimize the insonation condition in the specific target organs with various acoustic properties in larger animals [11].

Therefore, we chose to inject the plasmid into the myocardium rather than the peripheral vein or the LV chamber. In our study, we found that the combined therapy of direct intramyocardial injection of low-dose of HGF/MB and insonation induced more gene expression and capillary formation than either therapy alone. We analyze the reason as following: (1) Ultrasound can produce a variety of nonthermal bioeffects via acoustic cavitation and enhance the biological effects of microbubbles in the regional myocardium. The plasmids can take advantage of increased cell and capillary permeability after ultrasound-targeted microbubble destruction [11-14]. Thereby facilitating HGF DNA transfer through both artificial and biological membranes and enter cells in deeper layers [11-16]; thus a combination of approaches (direct administration of HGF and microbubbles into the myocardium and the use of US) resulted in high HGF expression in the HGF+MB/US group. The higher expression of HGF in the regional myocardium facilitates dramatic capillary growth. (2) Some studies have shown that ultrasonic destruction of microbubbles in the microcirculation causes local inflammatory cell infiltration, therefore inducing angiogenesis [30,31]. In our study, the ultrasonic destruction of microbubbles in the regional myocardium also direct induced angiogenesis.

As we mentioned before, invisible intramyocardial delivery could be dangerous in vivo, especially because myocardium cells may be replaced by scar tissue after myocardial infarction, and thus delivery of the gene could result in pericardial effusion or other injuries [32]. Evaluating the injection depth and breadth is critical for intramyocardial injection. Our study has shown that direct intramyocardial injection of microbubbles and a gene presents advantages for direct visualization. The microbubble-enhanced ultrasound imaging improves delineation of the injection border in the regional myocardium, which helps in estimating injection depth and radius in the ventricular wall. Moreover, left ventricular perforation and the intramyocardial injection of procedure can be monitored using echocardiography. This offers the potential advantage of accurately guiding transcatheter-targeted intramyocardial delivery of therapeutic agents.

The microbubble-enhanced imaging regions in the myocardium can be observed for 60 min. This long-lasting imaging time provides a good opportunity to observe the trace of the cell or gene mixed with microbubbles in the myocardium. Thus, intramyocardial injection of an extracellular microbubble contrast medium may be useful as a marker for observing the distribution of intramyocardial-delivered therapeutic agents.

Moreover, ultrasound can be focused and therefore targeted toward specific and, if necessary, deep locations within the body. If the biologic and clinical importance of angiogenic treatment is ultimately demonstrated, the method we describe in this report may constitute an important new strategy for treating patients with myocardial ischemic syndromes.

## Conclusions

In sum, systemic administration method need a high dose of gene and microbubble and induce side effect of non-target organ delivery. Direct intramyocardial injection of a mixture of a low-dose of an angiogenic gene with microbubbles followed by insonation treatment can enhance gene expression and angiogenesis. Microbubbles can also be used as a marker for observing the trace of the gene mixed with microbubbles in the myocardium, thus significantly increasing the safety of the intramyocardial injection procedure.

### Study limitations

Further investigations are needed to optimize this interesting, visible, effective, and novel gene delivery method. The gene expression pattern of EGFP and HGF were not tested in this study in order to reduce the number of dogs used. The efficiency of gene transfection by US/MB should be dependent upon insonation conditions as well as MBs structure. Therefore, further studies are necessary to optimize the insonation condition, and future studies should pursue the tradeoff between the efficacy and safety in respect to MB concentration and US intensity.

## Authors' contributions

YQY carried out the experiments, wrote the manuscript. HJ participated in the design of the study and helped to draft the manuscript. CBC participated in writing the manuscript. LXS participated in the experiments. SLY participated in writing the manuscript and the statistical analysis. All authors read and approved the final manuscript.
